# Health-related factors among seafarers based on the health ecology model: a cross-sectional study

**DOI:** 10.3389/fpubh.2026.1942282

**Published:** 2026-10-15

**Authors:** Dongdong Wu, Shilian Chen, Huaiyu Zheng, Xinwei Yu, Boer Yan

**Affiliations:** 1 Department of Pain Medicine, Zhoushan Hospital, Wenzhou Medical University, Zhoushan, Zhejiang, China; 2 Department of Neurology, Zhoushan Hospital, Wenzhou Medical University, Zhoushan, Zhejiang, China; 3 Department of Pharmacy, Zhoushan Hospital, Wenzhou Medical University, Zhoushan, Zhejiang, China; 4 Department of Hospital Administration, Zhoushan Hospital, Wenzhou Medical University, Zhoushan, Zhejiang, China; 5 Department of Nursing, Zhoushan Hospital, Wenzhou Medical University, Zhoushan, Zhejiang, China

**Keywords:** cross-sectional study, health ecological model, health status, influencing factors, seafarers

## Abstract

**Objective:**

To explore the health status and its influencing factors among seafarers based on the Health Ecological Model, and to identify priority areas for health management.

**Methods:**

A convenience sampling approach was used to administer a questionnaire survey to 106 seafarers from March to June 2026. The questionnaire was developed based on the Health Ecological Model and covers general information, health status, health-influencing factors, and health management needs. Multiple linear regression analysis was performed using SPSS 26.0.

**Results:**

The total health score was 29.65 ± 3.62 (theoretical range: 2–32). Policy environment (*β* = 0.602, *p* < 0.001) and behavioral characteristics (*β* = 0.260, *p* = 0.015) were independent influencing factors of the total health score, with an adjusted R2 of 0.574. Further analysis revealed that the policy environment had the greatest impact on physical health (*β* = 0.526, *p* < 0.001), while the interpersonal network was selectively associated with mental health (*β* = 0.207, *p* = 0.030). The top three health management needs were improving onboard dietary structure (87.7%), strengthening occupational health training (35.9%), and upgrading onboard medical facilities (31.1%).

**Conclusion:**

The overall health status of seafarers was at a relatively good level, with policy environment and behavioral characteristics being the main influencing factors. Health management should prioritize improving health security policies and onboard dietary structure, while strengthening occupational health training.

## 1 Introduction

The shipping industry is the lifeblood of global trade, with approximately 1.9 million seafarers sustaining over 90% of international trade transport (1). However, this occupational group faces health risks far exceeding those of land-based occupations, characterized by prolonged social isolation at sea, disrupted circadian rhythms, limited access to medical care, and confined living spaces—all of which constitute a unique constellation of health threats (2). The 2025 Gard Crew Claims Report indicates that seafarer death claims have increased by 25% in recent years, with disease-related deaths accounting for 83% and suicide rates surpassing accidental fatality rates (3). Epidemiological evidence has consistently demonstrated significant clustering of cardiovascular, metabolic, sleep, and mental health disorders among seafarers (4–6). However, the relative contributions of individual behaviors, interpersonal support, working conditions, and policy environments to seafarers’ health remain unquantified, leaving the prioritization of health interventions without empirical grounding.

Previous studies on seafarer health have been fragmented, predominantly focusing on single dimensions. Baygi et al. (7) employed a mixed-methods design and identified sleep abnormalities and lack of social interaction as important contributors to seafarer psychosocial health; however, the study did not incorporate working conditions or organizational policies into the analytical framework, implicitly assuming that individual-level interventions suffice—an assumption at odds with the structural realities of seafarer health problems. Qi et al. (8) examined the pathway linking loneliness to work engagement among Chinese seafarers, yet their perspective remained limited to the interpersonal psychological domain. Mampilli et al. (9) assessed the quality of life among Indian seafarers using the WHOQOL-BREF and found the lowest scores in the physical domain and the highest in the psychological domain, but did not systematically parse its multi-level determinants. Cui et al. (10) examined the epidemiological patterns and occupational predictors of electrocardiographic abnormalities in seafarers through a multicenter health screening analysis, but did not systematically parse its multi-level determinants. Although these studies have individually addressed associations between specific layers and health outcomes, they have failed to provide direct evidence on the priority ordering of interventions across different levels—a practical challenge confronting health decision-makers in shipping companies. Epidemiological research on Chinese seafarers has similarly concentrated on descriptive reports of disease prevalence (11, 12) or evaluations of single-behavior interventions (13), with no study to date simultaneously quantifying the relative contributions of behavioral, interpersonal, environmental, and policy-level factors within a unified theoretical framework. These limitations leave shipping companies unable to determine, under constrained health budgets, whether to prioritize improvements in onboard diet and physical activity conditions, or to enhance health security policies and medical examination systems—resource allocation thus lacks cross-level comparative evidence.

The Health Ecological Model (HEM) offers a systematic theoretical tool to address these issues. Rooted in Bronfenbrenner‘s ecological systems theory, the model posits that health is the product of interactions across five levels: individual traits, behavioral characteristics, interpersonal networks, living and working conditions, and policy environments (14). McLeroy et al. (15) introduced the ecological perspective into health promotion in 1988, highlighting the limitations of single-level interventions. However, HEM faces operationalization challenges in practice: although the five-level framework is widely cited, standardized approaches to measurement indicators, level delineation, and statistical modeling strategies for specific occupational groups remain lacking (16). The present study operationalized HEM specifically for the seafaring population, defining individual traits as age, gender, and history of chronic disease; behavioral characteristics as dietary patterns, physical activity, smoking status, and sleep duration; interpersonal networks as perceived onboard social support and satisfaction with peer relationships; working and living conditions as cabin space, noise exposure, and accessibility to medical care; and policy environments as health insurance coverage, periodic medical examination systems, and availability of mental health services—thereby providing a reproducible measurement framework for HEM applications in seafarer occupational health.

China has the largest seafaring population in the world, with over 2 million registered seafarers as of 2024 (17). Zhoushan City in Zhejiang Province, as China’s largest entry port for pelagic self-caught aquatic products, accounts for more than one-third of the national pelagic catch (18). However, health infrastructure for seafarers in this region remains critically underdeveloped: among over 350 pelagic fishing vessels in Putuo District, Zhoushan, only 11 are equipped with onboard medical clinics (19). Although local authorities have launched maritime health service initiatives to improve medication safety (20), the lack of comparative evidence on intervention effects across ecological levels makes it difficult for policymakers to make evidence-based choices among health education, environmental improvement, and institutional safeguarding. With the joint issuance in 2024 of the “Notice on Further Promoting the Occupational Health Protection Action and Enhancing Workers’ Occupational Health Literacy” by the National Health Commission and seven other ministries, which sets forth specific requirements for occupational health training, health literacy monitoring, and intervention among key populations (21), shipping companies and health administration departments urgently need to clarify prioritization under resource-constrained conditions. The present study aims to provide cross-level comparative evidence to inform tiered allocation of intervention resources.

The present study employed self-rated health as the core outcome indicator for seafarer health. Self-rated health has been extensively validated as an effective reflection of overall health status and a predictor of morbidity and mortality (22), with its reliability and predictive validity for mortality risk also established in the Chinese population (23). It has been utilized to identify health disparities across different groups within seafaring populations (24). Guided by the five-level framework of the Health Ecological Model, the present study proposes the following hypotheses: all five ecological dimensions exert independent explanatory effects on seafarer self-rated health; among these, behavioral characteristics and policy environment may demonstrate stronger explanatory power; and the contribution of interpersonal networks to mental health outcomes may exceed that to physical health. By quantifying the relative contributions of each dimension through multiple regression analysis, this study will provide evidence-based guidance for priority-setting in health intervention decisions under constrained health budgets.

## 2 Subjects and methods

### 2.1 Study design

This was a cross-sectional survey study, and its reporting followed the Strengthening the Reporting of Observational Studies in Epidemiology (STROBE) statement (25).

### 2.2 Study participants

Using a convenience sampling method, we distributed questionnaires to seagoing seafarers waiting for service at maritime service counters in Zhoushan City. The survey was conducted from March 1 to June 30, 2026. Uniformly trained research assistants explained the purpose of the survey and the confidentiality principles to the seafarers in the waiting areas. They invited them to participate voluntarily by scanning a QR code. This sampling strategy utilized the waiting time of seafarers who were handling maritime affairs such as certificate renewal, crew registration, and entry/exit procedures at the service counters, and thereby covered seafarers from various shipping companies, route types, and job categories, yielding good occupational heterogeneity.

Inclusion criteria: (1) currently employed seagoing seafarers; (2) had experience of sailing at sea; (3) voluntarily participated in this study and confirmed informed consent by clicking the “agree” button after reading the informed consent statement. Exclusion criteria: (1) incomplete questionnaire responses; (2) excessively short completion time (<180 s), which was considered invalid.

The selection of the survey site was carefully considered to ensure that the sample could cover a broad and diverse seafarer population. Maritime service counters are a mandatory venue for seafarers to complete occupation-related administrative procedures. Compared with other recruitment channels, such as distribution within a single shipping company, this channel could simultaneously reach seafarers on ocean-going and coastal routes, in management-level and operational-level positions, and employed by different shipping companies, thereby effectively reducing selection bias caused by the characteristics of a single company or a single route. At the same time, surveying during service waiting times enabled data collection without imposing an extra burden on seafarers’ normal schedules, balancing survey accessibility and participant convenience. This targeted site-sampling strategy aimed to obtain a sample with high occupational heterogeneity, thereby more comprehensively reflecting the health status and its ecological determinants among seagoing seafarers in the Zhoushan area.

### 2.3 Sample size estimation

A total of 109 questionnaires were distributed, and 107 were returned, yielding a response rate of 98.17%. After excluding 1 invalid questionnaire, 106 valid questionnaires were included, resulting in an effective response rate of 97.25%.

Regarding sample size adequacy, according to the rule of thumb for multiple linear regression analysis, the sample size should be at least 10–15 times the number of independent variables (26). In this study, 7 independent variables, combining continuous and categorical variables, were ultimately entered into the regression model. According to the minimum 10-times criterion, 70 cases were required; according to the more stringent 15-times criterion, 105 cases were required. The actual effective sample size of 106 cases met the minimum sample size requirement for statistical analysis and helped ensure the stability of the regression model’s estimates. We emphasize that the 10–15-times rule addresses the stability of parameter estimation rather than statistical power, and no formal *a priori* power calculation was performed. A sensitivity (*post hoc*) power analysis for the final model (*n* = 106, seven predictors, *α* = 0.05, two-tailed) indicated approximately 80% power to detect an effect of *f*^2^ ≥ 0.076 for an individual predictor (standardized *β* ≈ 0.27) and *f*^2^ ≥ 0.145 for the overall model (*R*^2^ ≈ 0.13). Effects below these thresholds could not be reliably detected. The sample was therefore adequate for estimating the primary model and detecting moderate-to-large effects, but underpowered for small effects; the interpretation of findings is stratified accordingly in the Discussion.

### 2.4 Research instruments

Data were collected using a self-developed Health Status Questionnaire for Seagoing Seafarers. The questionnaire was framed by the health ecology model (14) and incorporated the special occupational exposures of seagoing seafarers, such as prolonged voyages, shift work, and unbalanced nutrition. The initial draft was developed after literature review and two rounds of expert discussion. It was then revised for item wording and response time through a pilot survey (*n* = 20) to form the final version. The questionnaire consisted of four parts: the General Information Questionnaire, the Self-rated Health Status Scale, the Health Influencing Factors Scale, and the Health Management Needs Survey.

#### 2.4.1 General information questionnaire

This was self-designed and included 10 items: sex, age, educational level, years of service, job category, route area, family history of genetic diseases, smoking, alcohol consumption, and level of first-aid knowledge. Age was recorded as actual years, and the remaining items were measured at categorical levels. The variables in this section were mainly used to describe the baseline characteristics of the participants, among which age, educational level, and level of first-aid knowledge were entered as control variables in subsequent regression models.

#### 2.4.2 Self-rated health status scale

This scale was designed to assess the overall health status of seagoing seafarers. It consisted of 8 items across two dimensions: 6 on physical health and 2 on mental health. The physical health dimension included self-rated physical fitness, hearing status, number of chronic diseases, number of somatic discomfort symptoms in the past 6 months, number of past medical conditions, and number of occupation-related nutritional deficiencies. The mental health dimension included psychological status during voyages and depressed/anxious mood in the past 6 months.

All items were positively scored. Ordinal items, including self-rated physical fitness, hearing status, and psychological status during voyages, were assigned scores from 4 points (very good/normal) to 1 point (poor/severe abnormality). Dichotomous items were coded as 4 points for the absence of depressed or anxious mood in the past 6 months and 1 point for its presence. For multiple-choice items, such as chronic diseases, somatic discomfort symptoms, past medical conditions, and nutritional deficiencies, scoring was based on the number of options selected: 0 items selected received 4 points, 1 item received 2 points, and 2 or more items received 0 points. This scoring scheme was designed to reflect a dose–response gradient in health risk: absence of conditions (optimal health) received the maximum score (4), presence of a single condition indicated suboptimal health (2), and accumulation of two or more conditions represented substantial health impairment (0). The non-linear weighting (4–2-0 rather than 4-3-2-1-0) was chosen to emphasize the cumulative burden of multiple morbidities, which is a stronger predictor of health decline than the presence of any single condition. Littlejohns et al. (27) demonstrated a clear dose–response association between the number of co-occurring chronic conditions and all-cause mortality over 16 years of follow-up in a cohort of 502,370 adults, with particularly strong dose–response gradients for cardiovascular and respiratory deaths. This accelerating risk pattern supports the use of a categorical scoring system that assigns disproportionately lower scores to multiple morbidities, reflecting the non-linear deterioration of health status as disease burden accumulates.

The total health score was computed as the sum of all item scores, with a theoretical range of 2 to 32 points; higher scores reflected better health status. The physical and mental health dimension scores, ranging from 0 to 24 and 2 to 8 points, respectively, were also calculated for use in dimension-specific subgroup analyses. The scale demonstrated good internal consistency in this study, with a Cronbach’s *α* coefficient of 0.793.

The psychometric properties of the scale were further examined in this sample. The correlation matrix was factorable (Kaiser-Meyer-Olkin measure = 0.804; Bartlett’s test of sphericity: *χ*^2^ (28) = 239.013, *p* < 0.001). Parallel analysis and principal axis factoring supported a single dominant factor underlying all eight items (loadings 0.42–0.82; 35.8% of variance explained; McDonald’s *ω* = 0.811). We also examined the intended two-factor structure (6 physical + 2 mental items): although the two-factor solution converged (promax rotation; factor correlation = 0.70), it did not recover the intended item partition—the first factor combined the two mental items with hearing status, and the second combined chronic disease, somatic symptoms, past illness, and nutritional deficiencies (Supplementary Table 6). A confirmatory comparison showed that a two-factor model fitted the data better than a one-factor model, but the two factors were highly correlated (*r* = 0.83), indicating limited discriminant validity between the physical and mental subscales; the mental subscale, comprising only two items, could not be independently identified. Corrected item-total correlations ranged from 0.382 to 0.698, and no item would increase *α* if deleted. Sensitivity analyses showed that the total score was essentially unchanged under alternative scoring of the multiple-choice items (Spearman *r* = 0.9998 for linear scoring; 0.9968 for dichotomous scoring). The total score was therefore used as a composite index of self-rated health, whereas the physical and mental subscale scores were analyzed only as exploratory secondary outcomes.

#### 2.4.3 Health influencing factors scale

This scale was developed based on the health ecology model to measure the multi-level factors affecting the health of seagoing seafarers. It comprised 5 dimensions: individual characteristics, behavioral characteristics, interpersonal network, living and working conditions, and policy environment, with a total of 40 items/variables. The individual characteristics dimension consisted of 5 variables, namely age, educational level, job category, route area, and level of first-aid knowledge, derived from the General Information Questionnaire. These variables were formative indicators; they did not exhibit the covariance assumed by the theoretical framework and thus did not satisfy the prerequisite for testing Cronbach’s *α*. Therefore, they were not subjected to internal consistency testing and were included in the regression models as control variables at their original values. The four dimensions of behavioral characteristics, interpersonal network, living and working conditions, and policy environment were all reflective indicators. After uniform positive scoring, the item scores in each dimension were summed to obtain the dimension total score; higher scores indicated that the dimension level was more favorable to health. The Cronbach’s *α* coefficients for these four dimensions were 0.768, 0.643, 0.820, and 0.869, respectively. Except for the interpersonal network dimension, which was 0.643 (marginally acceptable and slightly below the conventional 0.70 threshold), the other dimensions reached good levels (*α* > 0.70), indicating acceptable internal consistency among items within each dimension (28). Unidimensionality of each reflective dimension was further supported by factor analysis: Bartlett’s test of sphericity was significant for all four dimensions (all *p* < 0.001), KMO values ranged from 0.718 to 0.818, and each dimension showed a single dominant factor with item loadings of 0.22–0.90, McDonald’s *ω* of 0.675–0.878, and variance explained of 34.8–64.5%, consistent with the Cronbach’s *α* values reported above (Supplementary Table 7). It should be noted that the policy environment dimension was operationalized as seafarers’ perceived policy environment—comprising their awareness of health-protection policies, the perceived arrangement and satisfaction of company medical check-ups, and willingness to participate in health interventions—rather than as objective policy indicators obtained from company or regulatory records.

#### 2.4.4 Health management needs survey

This was a single multiple-choice question with 8 options, designed to investigate seafarers’ willingness to prioritize needs regarding improving onboard dietary structure, strengthening occupational health training, upgrading onboard medical facilities and drug supplies, improving the psychological counseling system, optimizing route scheduling, establishing health records for seagoing seafarers, and enhancing family health communication, in order to understand seafarers’ priority demands for health management. The specific content of the questionnaire is presented in Appendix 1; the detailed items, scoring rules, and calculation methods for each dimension are presented in Appendix 2.

### 2.5 Data collection process

An electronic questionnaire was generated through Wenjuanxing, an online survey platform. The first page of the questionnaire contained an informed consent statement detailing the study purpose, data use, anonymity, and the right to withdraw voluntarily; participants could proceed only after checking the “agree” box. Uniformly trained research assistants explained the study’s purpose and confidentiality principles to seafarers in the waiting areas of the maritime service counters and invited them to participate voluntarily. Participants completed the questionnaire online by scanning the QR code. The platform automatically recorded the start and end times of questionnaire completion; responses with completion times <180 s were considered invalid and excluded. The researchers anonymized and coded the questionnaire data retrieved from the platform; after export, only coded variables were retained, with no personally identifiable information, and then statistical analyses were performed.

### 2.6 Statistical analysis

Data were analyzed using SPSS 26.0. Continuous variables were first tested for normality using the Kolmogorov–Smirnov test; those following a normal distribution were described as mean ± standard deviation (*x̄*± *s*), and those not following a normal distribution as median (interquartile range). Categorical variables were described as frequencies (*n*) and percentages (%). Cronbach’s *α* coefficient was used to assess the internal consistency of each reflective dimension. In univariate analyses, with the total health score as the dependent variable, an independent-samples t-test was used for dichotomous variables (route area); one-way ANOVA was used for polytomous variables (educational level, job category, and level of first-aid knowledge) when variances were homogeneous, and Welch’s correction was applied when variances were unequal; Pearson correlation analysis was used for continuous variables (age and scores on the four dimensions of the Health Influencing Factors Scale). The screening criterion was set at *p* < 0.10 for inclusion in subsequent multivariable models. Multivariable analysis was performed using forced-entry (Enter) multiple linear regression, with all variables with *p* < 0.10 in univariate analyses entered simultaneously into the regression equation. Model evaluation included the overall F test, coefficient of determination (*R^2^*), adjusted *R^2^*, collinearity diagnostics (variance inflation factor <10), and residual independence test (Durbin–Watson statistic close to 2). To further explore the differential effects of variables on different health dimensions, separate multiple linear regression analyses were performed using the same set of independent variables, with the physical health dimension score and mental health dimension score as dependent variables, respectively. For effect sizes, partial *η*^2^ was reported for ANOVA, and standardized regression coefficients (*β*) and *R^2^* change were reported for regression analyses. All tests were two-tailed, and *p* < 0.05 was considered statistically significant. 95% confidence intervals (CIs) were reported for all regression coefficients.

### 2.7 Ethical considerations

This study was approved by the Ethics Committee of Zhoushan Hospital (approval No. 2026–003-01). All participants had already read the informed consent statement online before the survey and confirmed voluntary participation by clicking the “agree” button, in accordance with ethical norms for electronic informed consent. The questionnaire was anonymous, backend data were accessible only to the researchers, and all personally identifiable information was removed during data processing to protect participant privacy strictly. The data were used solely for academic research and were properly archived in accordance with relevant data protection regulations.

## 3 Results

### 3.1 Basic characteristics of the study population

A total of 106 valid cases were included in this study, all of whom were male. The mean age of the participants was 48.39 ± 10.43 years. Regarding educational level, junior high school was the most common (49.06%). In terms of years of service, more than 10 years accounted for the majority (65.09%). The most frequent job category was management level (45.28%), and the main service area was coastal waters (74.53%). For first-aid knowledge, the majority reported basic knowledge (49.06%). Other demographic characteristics are presented in Table 1.

**Table 1:** General information of seafarers (*n* = 106).

Variable	Category	*n (%)*/*x̄* ± *s*
Age (years)	—	48.39 **±** 10.43
Educational level	Primary school or below	9 (8.49)
	Junior high school	52 (49.06)
	Senior high/technical secondary	23 (21.70)
	College degree or above	22 (20.75)
Years of service	≤3 years	14 (13.21)
	3–5 years	9 (8.49)
	5–10 years	14 (13.21)
	>10 years	69 (65.09)
Job category	Management level	48 (45.28)
	Operational level	26 (24.53)
	Support level	32 (30.19)
Service area	Coastal waters	79 (74.53)
	Ocean‑going	27 (25.47)
Family history of hereditary disease	Yes	4 (3.77)
	No	102 (96.23)
Smoking	Yes	59 (55.66)
	No	47 (44.34)
Alcohol consumption	Yes	29 (27.36)
	No	77 (72.64)
First‑aid knowledge	Very proficient	47 (44.34)
	Basic knowledge	52 (49.06)
	Limited	7 (6.60)
	No knowledge	0 (0)

All 106 seafarers in this study were male, so gender is not listed in the table. Age is presented as mean ± standard deviation; all other variables are presented as n (%).

### 3.2 Scores of health status and health-influencing factors in each dimension

The total health score and scores of each dimension of health-influencing factors are shown in Table 2. In this group, the total health score was 29.65 ± 3.62 (theoretical range: 2–32; observed range: 13–32), indicating a moderately high level overall. The physical and mental health dimension scores were 22.27 ± 2.67 and 7.38 ± 1.35, with theoretical ranges of 0–24 and 2–8, respectively. Further Pearson correlation analysis showed a significant positive correlation between the physical health dimension and the mental health dimension (*r* = 0.577, *p* < 0.01), indicating that the two subscales are interrelated; however, given their limited discriminant validity in this sample (Section 2.4.2), their separate analysis should be regarded as exploratory.

**Table 2:** Scores of health status and health‑influencing factors in each dimension (*n* = 106).

Variable	Number of items	Theoretical range	Observed range	*x̄*±*s*
Dependent variables				
Total health score	8	2–32	13–32	29.65 ± 3.62
Physical health dimension	6	0–24	11–24	22.27 ± 2.67
Mental health dimension	2	2–8	2–8	7.38 ± 1.35
Independent variables				
behavioral characteristics	6	0–21	11–21	18.10 ± 2.75
Interpersonal network	4	0–12	4–12	9.20 ± 1.52
Living and working conditions	11	0–37	18–37	29.30 ± 5.35
Policy environment	4	1–16	4–16	13.08 ± 3.00

Among the dimensions of health-influencing factors, the behavioral characteristics score was 18.10 ± 2.75 (theoretical range 0–21), the interpersonal network score was 9.20 ± 1.52 (theoretical range 0–12), the living and working conditions score was 29.30 ± 5.35 (theoretical range 0–37), and the policy environment score was 13.08 ± 3.00 (theoretical range 1–16). Pearson correlation analysis among the four influencing-factor dimensions showed that all dimensions were positively correlated (*r* range: 0.547–0.727, *p* < 0.01; Supplementary Table 1), with the highest correlation between behavioral characteristics and working conditions (*r* = 0.727). Although associations exist among the dimensions, they were within acceptable ranges.

### 3.3 Univariate analysis of Total health score

The results of univariate analysis are shown in Table 3. Pearson correlation analysis revealed that age (*r* = 0.195, *p* < 0.05) was weakly positively correlated with the total health score. Behavioral characteristics score (*r* = 0.643, *p* < 0.01), interpersonal network score (*r* = 0.527, *p* < 0.01), living and working conditions score (*r* = 0.543, *p* < 0.01), and policy environment score (*r* = 0.739, *p* < 0.01) all showed moderate-to-strong positive correlations with the total health score. There was no statistically significant difference in total health score between coastal and ocean-going seafarers (*t* = 0.950, *p* = 0.344, Cohen’s *d* = 0.21). For multi-category variables: job category had homogeneous variances (Levene’s test, *p* > 0.05); one-way ANOVA indicated no significant differences among groups (*F* (2, 103) = 0.924, *p* = 0.400, partial *η*^2^ = 0.018). Educational level and first-aid knowledge had unequal variances; Welch’s ANOVA showed statistically significant overall differences (*F* (3, 35.3) = 4.572, *p* = 0.008, partial *η*^2^ = 0.280; *F* (2, 16.7) = 11.355, *p* < 0.001, partial *η*^2^ = 0.577, respectively), and Games-Howell post-hoc comparisons revealed significant differences between certain groups.

**Table 3:** Univariate analysis of total health score by different characteristics (*n* = 106).

Variable	Category	*n* (%)	Total health score (*x̄*± *s*)	*t*/*F*/*r*	Effect size
Age (years)		106		*r*=0.195*	*r*^2^ = 0.038
Educational level	Primary school or below	9 (8.49)	31.22 ± 1.31	*F*=4.572**	partial *η*² = 0.280
	Junior high school	52 (49.06)	30.62 ± 2.44		
	Senior high/ technical secondary	23 (21.70)	27.87 ± 5.00		
	College degree or above	22 (20.75)	28.59 ± 4.01		
Job category	Management level	48 (45.28)	29.38 ± 3.51	*F*=0.924	partial *η*² = 0.018
	Operational level	26 (24.53)	29.27 ± 3.83		
	Support level	32 (30.19)	30.38 ± 3.61		
Service area	Coastal waters	79 (74.53)	29.46 ± 3.75	*t*=0.950	*d* = 0.21
	Ocean‑going	27 (25.47)	30.22 ± 3.20		
First‑aid knowledge	Very proficient	47 (44.34)	31.17 ± 1.80	*F*=11.355***	partial *η*² = 0.577
	Basic knowledge	52 (49.06)	28.46 ± 4.42		
	Limited	7 (6.60)	28.29 ± 2.36		
Behavioral characteristics score		106		*r*=0.643**	*r*² = 0.413
Interpersonal network score		106		*r*=0.527**	*r*² = 0.278
Living and working conditions score		106		*r*=0.543**	*r*² = 0.295
Policy environment score		106		*r*=0.739**	*r*² = 0.546

*p < 0.05, **p < 0.01, ***p < 0.001 (two‑tailed). For ANOVA, partial *η*² is reported as the effect size; for the t‑test, Cohen's *d* is reported; for Pearson correlations, *r* itself is the effect size, with r² indicating the proportion of variance explained.

### 3.4 Multivariate analysis of Total health score

Using total health score as the dependent variable, seven variables, namely age, educational level, first-aid knowledge, behavioral characteristics score, interpersonal network score, living and working conditions score, and policy environment score, were entered into a multiple linear regression model. The overall model test showed *F*(7,98) = 21.232, *p* < 0.001, indicating that the regression model was statistically significant. The coefficient of determination (*R^2^*) = 0.603 and the adjusted *R^2^* = 0.574, suggesting that the seven variables together explained 57.4% of the variance in the total health score. The Durbin-Watson statistic was 1.693, which lies between 1.5 and 2.5, indicating no first-order autocorrelation in the residuals and satisfying the independence assumption. Collinearity diagnostics showed tolerance values of 0.342–0.770 and VIF values of 1.298–2.928 for all independent variables, with a maximum condition index of 3.7, indicating that collinearity, although non-trivial, did not preclude stable estimation.

The regression results for each independent variable were as follows: the standardized regression coefficient for policy environment score was *β* = 0.602 (*t* = 5.947, *p* < 0.001), positively and significantly predicting total health score – higher policy environment scores were associated with higher total health scores. The standardized coefficient for the behavioral characteristics score was *β* = 0.260 (*t* = 2.473, *p* = 0.015), also positively and significantly predicting total health score – better behavioral characteristics were associated with better health status. Age, educational level, first-aid knowledge, interpersonal network score, and living and working conditions score did not show statistically significant effects on total health score (all *p* > 0.05). The detailed results of the multiple linear regression analysis are presented in Table 4.

**Table 4:** Multiple linear regression analysis of factors influencing total health score (*n* = 106).

Variable	*β*	SE	*Beta*	*t*	*p*	Tolerance	VIF
Constant	13.128	2.469		5.318	<0.001		
Age	0.035	0.025	0.100	1.375	0.172	0.770	1.298
Educational level^a^	0.046	0.299	0.012	0.153	0.879	0.706	1.417
First‑aid knowledge^b^	−0.299	0.448	−0.050	−0.667	0.506	0.714	1.400
Behavioral characteristics score	0.343	0.139	0.260	2.473	0.015	0.366	2.730
Interpersonal network score	0.300	0.196	0.125	1.527	0.130	0.600	1.666
Living and working conditions score	−0.092	0.074	−0.137	−1.254	0.213	0.342	2.928
Policy environment score	0.725	0.122	0.602	5.947	<0.001	0.396	2.524

Dependent variable is total health score; *β* (B) is unstandardized regression coefficient, Beta is standardized regression coefficient; VIF is variance inflation factor. Model: *F*(7,98) = 21.232, *p* < 0.001, *R²* = 0.603, adjusted *R²* = 0.574, Durbin‑Watson = 1.693.

aCoding for educational level: primary school or below = 1, junior high school = 2, senior high / technical secondary = 3, college degree or above = 4.

bCoding for first‑aid knowledge: no knowledge = 1, limited = 2, basic knowledge = 3, very proficient = 4.

Notably, these diagnostics indicate only that estimation was not impaired; given the substantial bivariate correlations among the four ecological dimensions (*r* = 0.547–0.727), they cannot demonstrate that the independent effects of each dimension have been cleanly separated. We therefore conducted two supplementary analyses. First, a leave-one-dimension-out analysis (Supplementary Table 4) showed that the standardized coefficient of the policy environment remained the largest in every model (*β* = 0.560–0.672) and that of behavioral characteristics remained positive (*β* = 0.214–0.437); in contrast, the coefficient for living and working conditions changed sign (−0.137 to +0.074) and that for the interpersonal network remained small (0.109–0.221), indicating that the unique effects of these two dimensions cannot be reliably separated from the shared variance. Second, a commonality analysis (Supplementary Table 5) showed that, of the four dimensions’ joint *R^2^* of 0.593, the unique contributions were small (0.004–0.135), whereas 61% of the summed zero-order contributions represented overlapping variance that cannot be attributed to any single dimension. These results indicate that the four ecological dimensions operate as an intercorrelated block, and the regression coefficients should be interpreted as conditional unique contributions rather than fully separated effects.

### 3.5 Supplementary analysis of factors influencing physical and mental health dimensions

To further explore the distinct effects of each ecological dimension on different aspects of seafarers’ health, we conducted separate regression analyses, using the physical and mental health dimensions as dependent variables and the same set of independent variables as in the main analysis. The results are presented in Supplementary Tables 2, 3. For the physical health dimension, the final model had an adjusted *R^2^* of 0.492, indicating that all independent variables together explained 49.2% of the variance in physical health. Among them, the behavioral characteristics score (*β* = 0.260, *p* = 0.022) and the policy environment score (*β* = 0.526, *p* < 0.001) were independent influencing factors for physical health. For the mental health dimension, the final model had an adjusted *R^2^* of 0.444. Age (B = 0.036 per year, 95% CI: 0.015–0.057; standardized *β* = 0.279, *p* = 0.001), interpersonal network score (B = 0.185, 95% CI: 0.019–0.351; *β* = 0.207, *p* = 0.030), and policy environment score (B = 0.199, 95% CI: 0.096–0.303; *β* = 0.444, *p* < 0.001) had significant independent effects on mental health. For age, although statistically significant, the absolute magnitude was modest: a one-standard-deviation difference in age (10.4 years) corresponded to approximately 0.37 points on the 2–8 point mental health scale (≈0.28 SD), and this finding should be regarded as exploratory given the limited power for small effects (see Section 2.3).

Comparing the two dimensions, the effect showed a differentiated pattern: behavioral characteristics influenced physical health (*β* = 0.267) but not mental health; the interpersonal network influenced mental health (*β* = 0.207) but not physical health (*β* = 0.065), with a larger standardized effect on mental health and the policy environment significantly affected both dimensions, with a larger standardized effect on physical health (*β* = 0.591) than on mental health (*β* = 0.444).

### 3.6 Analysis of health management needs of seafarers

Seafarers’ demands for health management were investigated. The top three demands were improving the diet on board (87.74%), strengthening occupational health training (35.85%), and upgrading onboard medical facilities and medicine supplies (31.13%). In addition, establishing seafarer health records for dynamic health management was selected by 18.87%, while improving mental health support systems, optimizing voyage scheduling, and enhancing communication with families each accounted for 17.92%, and others accounted for 0.94%. The results are shown in Figure 1.

**Figure 1:**
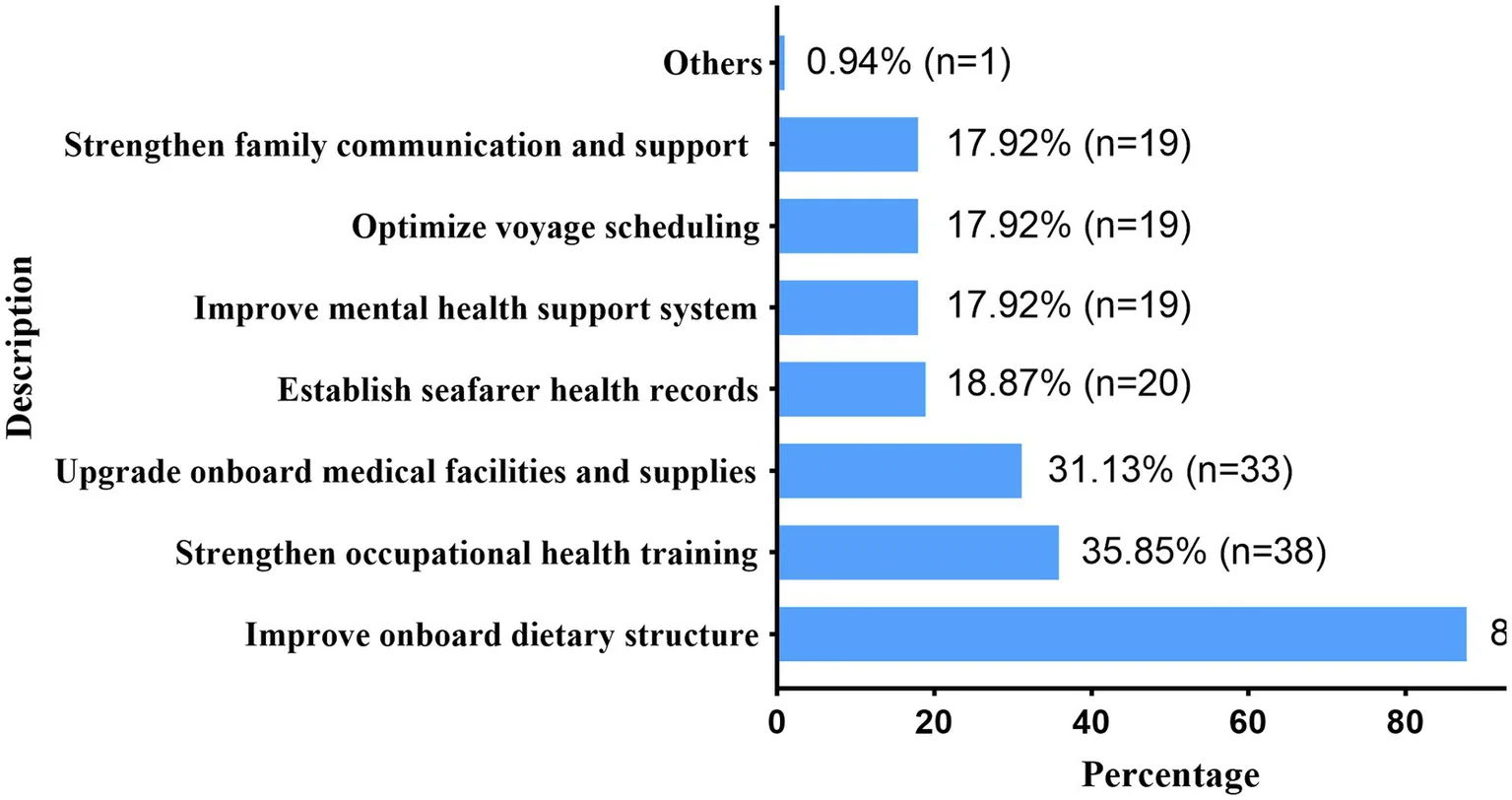
Distribution of health management needs among seafarers (*n* = 106).

## 4 Discussion

The total health score of seafarers in this group approached the theoretical maximum (29.65 ± 3.62/32 points), suggesting an overall good health status in this sample, which may be attributable to the predominance of management-level crew (45.28%) and coastal-route service (74.53%), as these groups likely enjoy better working conditions and living support compared with ocean-going ordinary seafarers. Previous studies have demonstrated that seafarers’ health levels are closely related to job responsibilities and working environment (24); however, in the present study, job category and service area did not show significant differences in health, possibly due to the relatively concentrated sample composition. Physical and mental health showed a moderate positive correlation (*r* = 0.577), indicating that the two are interrelated; however, given their limited discriminant validity (Section 2.4.2), the dimension-specific analyses should be regarded as exploratory.

Multiple regression analysis showed that the policy environment (*β* = 0.602) and behavioral characteristics (*β* = 0.260) were independent factors influencing the total health score, with the model explaining 57.4% of the variance. The policy environment was the most influential variable, encompassing health policy awareness, medical examination systems, and willingness to participate in health interventions (16). This finding suggests that macro-level health protection systems play a fundamental supporting role in seafarers’ health—regular health examinations facilitate early detection of health problems, and policy awareness may promote the adoption of health-promoting behaviors. Previous studies have rarely examined the impact of the policy environment dimension on seafarers’ health, and the present study fills this gap. Jiang et al. (29), using machine-learning-based analysis, also found that work-life conditions were the strongest determinant of seafarers’ mental health, thereby empirically validating the foundational role of the macro policy environment in seafarers’ health. The health ecological model emphasizes that the policy environment, as the outermost ecological system, exerts cross-level influences on individual health through resource allocation, institutional norms, and health service provision (14, 15). The dominant effect of the policy environment dimension in this study verifies this theoretical expectation. It suggests that shipping health management should not be limited to individual-level behavioral interventions, but should also build supportive environments at the institutional level (21). To examine whether this association merely reflects health awareness rather than the policy environment itself, we decomposed the dimension into an institutional component (regular check-up arrangement and satisfaction, Q42–Q43) and an awareness/willingness component (policy awareness and willingness to participate, Q41 and Q44). Both components independently predicted the total health score when entered simultaneously (*β* = 0.377, *p* < 0.001 and *β* = 0.262, *p* = 0.029, respectively), and the institutional component alone remained a strong predictor (*β* = 0.501, *p* < 0.001). Moreover, the primary model already adjusted for behavioral characteristics, which include the health-attention item, so the coefficient for the policy environment is net of measured individual health awareness. These results indicate that the association cannot be attributed solely to health awareness or response tendency, although the perceived nature of the measure warrants caution.

Behavioral characteristics, including diet, sleep, exercise, and health awareness, also significantly influenced health, consistent with previous findings (12). However, it is noteworthy that working conditions were significantly correlated with total health score in univariate analysis (*r* = 0.543) but did not reach statistical significance in the multivariate model, possibly due to collinearity among behavioral characteristics, policy environment, and working conditions (behavioral characteristics and working conditions: *r* = 0.727; policy environment and working conditions: *r* = 0.686), with the shared explanatory variance being absorbed by statistically more significant variables in the multiple regression (26). Given the substantial correlations among ecological-level predictors, future research should validate these pathways using structural equation modeling (SEM) to disentangle direct and indirect effects across ecological levels. The interpersonal network dimension, despite showing a moderate bivariate correlation with the total health score (*r* = 0.527, *p* < 0.01), did not retain significance in the multivariate model. Statistically, this disappearance likely reflects a suppression effect attributable to shared variance: the interpersonal network score was substantially correlated with both the policy environment (*r* = 0.668) and behavioral characteristics (*r* = 0.587). Once these more dominant predictors were entered into the regression equation, the unique contribution of interpersonal networks fell below the detection threshold, particularly given the limited sample size. Theoretically, the high mean score of interpersonal networks (9.20/12, theoretical range 0–12) suggests a ceiling effect, with restricted between-individual variability that reduced statistical power to detect an independent effect. This does not negate the theoretical importance of social support; rather, it indicates that in this relatively homogeneous and socially cohesive sample—predominantly management-level crew on coastal routes—interpersonal support may have been universally adequate, leaving insufficient variance to demonstrate an independent association after accounting for macro-level policy factors. This interpretation is corroborated by the supplementary analysis, in which interpersonal networks did significantly predict mental health (*β* = 0.185, *p* = 0.030), confirming that its effect is dimension-specific rather than absent. In other words, social support may operate primarily through psychological buffering pathways for mental health, while its contribution to overall health is largely redundant with policy-level and behavioral resources in this particular sample. Age showed a weak positive correlation with total health score in univariate analysis (*r* = 0.195, *p* < 0.05) but was not significant in the multivariate model, possibly because the wide age range partially masked the independent effect of age, or because behavioral characteristics and working conditions mediated the effect of age. Educational level and first-aid knowledge showed significant between-group differences in univariate analysis. However, they did not reach statistical significance in the multivariate model, these null results are inconclusive and may reflect limited power rather than the absence of an association after controlling for confounders.

The substantial intercorrelations among the four ecological dimensions also warrant caution when interpreting the relative magnitude of the coefficients. The leave-one-dimension-out analysis showed that the dominance of the policy environment was unaffected by which other dimension was removed (*β* = 0.560–0.672 across all models), supporting the robustness of our primary conclusion. However, the coefficient for living and working conditions reversed sign depending on model composition, and the commonality analysis indicated that the majority (61%) of the variance explained by the four dimensions was shared rather than unique. These findings suggest that the four dimensions are best understood as an intercorrelated ecological block whose members mutually reinforce one another—consistent with the health ecological model’s premise of cross-level interaction—rather than as four independent influences. Consequently, the standardized coefficients in Table 4 represent conditional, unique contributions, and the non-significance of living and working conditions and the interpersonal network should not be interpreted as evidence that these levels are unimportant; rather, their effects are largely redundant with the policy environment and behavioral characteristics in this sample.

Supplementary analyses further revealed differentiated pathways from each dimension to physical versus mental health: behavioral characteristics significantly influenced only physical health, suggesting that lifestyle factors such as diet, exercise, and sleep primarily operate through somatic metabolic and functional maintenance pathways, with limited direct effects on psychological status. Zhao et al. (30), through a simulated cabin experiment, found that optimized lighting significantly improved seafarers’ drowsiness and fatigue levels and extended sleep duration, suggesting that environmental factors can indirectly influence physical health by regulating sleep, which is consistent with the independent effect of behavioral characteristics on physical health in this study. Kerkamm et al. (31) also found that seafarers’ sleep quality was objectively impaired, with watchkeeping personnel being particularly affected, further supporting the key role of sleep as a mediator between behavioral characteristics and working conditions in the physical health pathway. The interpersonal network significantly influenced only mental health, suggesting that social resources such as colleague support and family understanding primarily operate through emotional regulation and psychological buffering pathways. Strukcinskiene et al. (32) also found that colleague and supervisor support were the main buffering factors against seafarers’ work-related stress, and that junior seafarers perceived significantly higher stress on interpersonal dimensions, supporting the protective role of social support on mental health during occupational adaptation. Seafarers work in a closed environment far from family (8); lack of social support may first manifest as psychological loneliness and anxiety. The policy environment was the only dimension that significantly influenced both physical health (*β* = 0.591) and mental health (*β* = 0.444). However, its effect size on physical health was much larger than on mental health, suggesting that macro-level measures such as medical examination systems and health policies primarily improve physical health through disease screening and prevention pathways. This finding, together with Jiang et al.’s (29) conclusion on the dominant effect of work-life conditions, jointly indicates that institutional-level health protection plays a fundamental supporting role for seafarers. Age was associated only with mental health and not with physical health in this study, although the effect was modest in absolute magnitude—a one-standard-deviation difference in age (10.4 years) corresponded to approximately 0.37 points on the 2–8 point scale (≈0.28 SD)—and should be regarded as exploratory given the limited power for small effects (Section 2.3). This pattern may be related to the predominance of coastal-route and management-level crew in the sample, as relatively stable work rhythms and environmental conditions may partially offset the physiological decline effects associated with aging,

The above literature collectively suggests that protective factors for seafarers’ health span a multi-level system encompassing individual behaviors, interpersonal relationships, and organizational environments, with possible interactive enhancement or compensatory effects among levels, further supporting the applicability of the health ecological model in seafarer populations (14, 15). These findings refine the application of the health ecological model to seafarers, revealing the distinct pathways through which ecological dimensions influence physical versus mental health, and providing empirical evidence to inform the development of stratified, targeted health intervention strategies for seafarers (16).

Seafarers’ demands for health management showed a pattern of “one dominant demand supported by multiple secondary demands.” “Improving the diet on board” was the highest demand (87.74%), echoing the finding that the behavioral characteristics dimension significantly influenced health, suggesting that dietary improvement is the most salient health need perceived by seafarers. Wang et al.’s (13) intervention study also demonstrated that personalized dietary nutrition knowledge-attitude-practice interventions effectively improved seafarers’ nutritional status, further validating the operability and practical value of dietary management in health promotion among seafarers. It is recommended that shipping companies increase the supply of fresh fruits, vegetables, and high-quality protein where conditions permit, and establish a scientific dietary rotation system. “Strengthening occupational health training” ranked second (35.85%), consistent with the significant association between first-aid knowledge and health in univariate analysis. Jensen and Oldenburg (33) also pointed out that stress management and cross-cultural communication training for seafarers are seriously insufficient in maritime higher education, and recommended incorporating stress management and diversity training into the routine education system for future seafarers. Demands such as “upgrading onboard medical facilities and medicine supplies” (31.13%) and “establishing health records” (18.87%) were consistent with the core influence of the policy environment dimension, suggesting that macro-level health protection measures need further implementation (20). Notably, the demand for “improving mental health support systems” was only 17.92%, which, combined with the relatively high mental health scores (7.38/8 points) in this group, may reflect that the overall mental health status of the sample was relatively good and psychological counseling had not yet become a widespread need. However, given the unique characteristics of the seafaring profession (5), continued attention to mental health remains necessary. In addition, this study found that interpersonal networks were significantly correlated with mental health, suggesting that, beyond dietary improvement and occupational training, psychological health may be indirectly promoted by building peer support systems and family collaborative support mechanisms among crew members.

Finally, we explicitly classify the robustness of our findings in light of the limited statistical power. (i) Well-supported conclusions: the policy environment was by far the strongest predictor of the total health score (*β* = 0.602, *f^2^* = 0.334), an effect sufficiently large that the achieved power was essentially 100%, so its dominance is unlikely to be a power artifact; the moderate-to-strong bivariate correlations of the four ecological dimensions with health (*r* = 0.527–0.739) are likewise robust. (ii) Significant but requiring confirmation: behavioral characteristics (*β* = 0.260, *p* = 0.015; *f^2^* = 0.058; achieved power ≈ 69%) lies close to the minimum detectable effect. (iii) Exploratory findings: the dimension-specific pathway analyses (behavioral characteristics → physical health; interpersonal networks and age → mental health) were based on smaller effects in a sample with limited power and were intended to generate rather than confirm hypotheses. (iv) Uncertain conclusions: the non-significant coefficients (interpersonal networks, living and working conditions, educational level, first-aid knowledge) and the null between-group differences (job category, service area) all lie below the minimum detectable threshold (|*β*| < 0.27) and therefore cannot be interpreted as evidence of no effect; they remain inconclusive and require re-examination in adequately powered samples.

The present study has several limitations. First, although the sample size (n = 106) satisfied the 10–15-times rule for regression stability, this rule does not guarantee statistical power: our sensitivity analysis (Section 2.3) showed that only effects of |*β*| ≥ 0.27 could be detected with 80% power. The null results for interpersonal networks and working conditions are therefore inconclusive rather than confirmatory, and the study was not designed to detect small effects; future studies should recruit larger, stratified samples. Second, the sample was drawn from a single region (Zhoushan) using convenience sampling, and its demographic composition diverged markedly from the broader seafaring workforce: all participants were male (100%), nearly three-quarters were on coastal routes (74.5%), and almost half held management-level positions (45.3%). This triple selection bias likely inflated the observed health scores and attenuated the effects of occupational stressors, because coastal management crew typically experience more regular schedules, better living conditions, and greater access to shore-based medical services than ocean-going ratings. Consequently, the findings may not generalize to the large population of operational-level seafarers on long-haul voyages, who face more severe physical and psychosocial exposures. Future studies should employ stratified random sampling to ensure proportional representation across gender, route types, and rank levels. Third, the cross-sectional design can only reveal correlational relationships among variables and cannot infer causal relationships (25); future longitudinal tracking or intervention studies are needed to validate further the pathways through which each ecological dimension influences health. Fourth, all health-related variables were collected through self-report questionnaires, which may be subject to recall and social desirability biases (28). Future research could incorporate objective health indicators, such as medical examination data and physiological measurements, to validate findings. Fifth, the sample comprised only male seafarers, precluding examination of gender differences in health ecological pathways. According to the Baltic and International Maritime Council and International Chamber of Shipping (34) report, women account for only about 2% of the global seafaring workforce, and male predominance is a global industry characteristic of shipping. Therefore, the gender composition of this sample reflects the industry reality to a certain extent. However, as the proportion of female seafarers increases in the future, gender-comparative studies will be necessary to test the applicability of the health ecological model across different gender groups. Sixth, the Cronbach’s *α* coefficient for the interpersonal network dimension (0.643) was marginally acceptable and fell slightly below the conventional 0.70 threshold. This relatively weak internal consistency may have increased measurement error, thereby attenuating the observed association between interpersonal networks and health outcomes and contributing to its non-significance in the multivariate model. Further research should refine the item design for this dimension, potentially by adding items related to family support, onboard peer cohesion, or shore-based social connectedness to improve reliability (28). Seventh, some variables, such as first-aid knowledge, showed skewed distributions, limiting the range of variation for these variables and the power to detect their effects on health (26). Eighth, as no control group was included in this study, direct comparison of the relative effectiveness of different intervention strategies was not possible. Follow-up studies could include seafarer groups from different regions (18) and different route types (17) for comparative analysis. Ninth, measurement limitations of the self-developed instruments should be acknowledged. The Self-rated Health Status Scale currently has only preliminary psychometric support: although internal consistency and a single dominant factor were demonstrated, the two-factor structure was not clearly confirmed, and test–retest reliability and convergent/discriminant validity against external criteria were not assessed. In addition, the policy environment dimension was measured by self-reported perceptions rather than objective policy indicators (e.g., company records of health insurance coverage or check-up systems), and common-method bias and social desirability in these items cannot be fully excluded. Future studies should validate the scales against objective health indicators, incorporate test–retest assessment, and collect company-level policy data.

## 5 Conclusion

Based on the health ecological model, this study examined the relative contributions of five dimensions—individual characteristics, behavioral characteristics, interpersonal networks, working conditions, and policy environment—to self-rated health among seafarers. The results showed that the total health score of seafarers was moderately high (29.65 ± 3.62 out of 32 points), with the policy environment (*β* = 0.602) and behavioral characteristics (*β* = 0.260) as the primary influencing factors. The effects of each dimension on physical and mental health exhibited pathway specificity: behavioral characteristics predominantly acted on physical health, interpersonal networks mainly affected mental health, and the policy environment had significant effects on both. These findings refine the application of the health ecological model to seafarers and reveal distinct pathways for interventions across ecological levels. It is recommended that health management prioritize improving health protection policies, the onboard dietary structure, and onboard medical facilities, strengthening occupational health training, and establishing mutual support systems among crew members and family collaborative support mechanisms. Future studies should adopt longitudinal designs to validate causal relationships, expand samples to cover ocean-going routes and various ranks, and include more multi-level variables, such as organizational culture, to comprehensively reveal the socio-ecological determinants of seafarers’ health.

## Data Availability

The raw data supporting the conclusions of this article will be made available by the authors, without undue reservation.

## Supplementary material

Supplementary Table 1 Correlation analysis of scores of health-influencing factor dimensions.

Supplementary Tables 2/3 Multiple linear regression analysis of factors influencing the physical/mental health dimension score.

Supplementary Table 4 Coefficient stability in leave-one-dimension-out models.

Supplementary Table 5 Commonality analysis of the four ecological dimensions.

Supplementary Table 6 Psychometric evaluation of the self-rated health status scale.

Supplementary Table 7 Factor analysis of the four reflective dimensions of the health influencing factors scale.

Appendix 1 Health survey questionnaire for seafarers.

Appendix 2 Scoring rules for variables.

Appendix 3 STROBE statement checklist.
