# Supplementary material for: Health-related factors among seafarers based on the health ecology model: a cross-sectional study
Source: Front Public Health. 2026 Oct 15;14:1942282. doi: 10.3389/fpubh.2026.1942282 (PMC13716717; doi:10.3389/fpubh.2026.1942282)
Supplement: Supplementary file 1 [file Supplementary_file_1.docx]

**Appendix 1: Health Survey Questionnaire for Seafarers**

Dear Seafarer,

This survey aims to understand seafarers' health status and health management needs. It is intended for scientific research purposes only. The questionnaire is anonymous, and all data will be kept strictly confidential and used solely for overall statistical analysis; no personal information will be disclosed. Participation is entirely voluntary, and you may refuse to answer or stop filling out the questionnaire at any time without any negative consequences. It will take approximately 10–15 minutes to complete. By completing this questionnaire, you indicate that you have fully understood the above information and voluntarily agree to participate in this study.

1. Questionnaire ID: ______

**Part I: General Information of Seafarers**

2. Gender:

A. Male B. Female

3. Age: _____ years old

4. Highest level of education:

A. Primary school or below B. Junior high school C. Senior high school / technical secondary school (including maritime vocational schools) D. College degree or above

5. Years of seafaring experience:

A. 3 years or less B. 3–5 years C. 5–10 years D. More than 10 years

6. Rank/job category:

A. Management level (Captain, Chief Officer, Chief Engineer, Second Engineer)

B. Operational level (Second Officer, Third Officer, Third Engineer, Fourth Engineer)

C. Support level (Able Seaman, Oiler, Cook, Purser, etc.)

7. Main service area:

A. Coastal waters B. Ocean‑going

8. Do you have a family history of hereditary diseases (e.g., cardiovascular disease, diabetes, mental disorders, etc.)?

A. Yes (please specify: ______) B. No

9. Smoking habit:

A. Yes B. Former smoker, quit within the last 6 months C. Former smoker, quit more than 6 months ago D. No

10. Alcohol consumption:

A. Yes B. Former drinker, quit within the last 6 months C. Former drinker, quit more than 6 months ago D. No

11. How would you rate your knowledge of first aid?

A. Very proficient – able to independently perform first aid for common emergencies (e.g., heatstroke, trauma, cardiac arrest)

B. Basic knowledge – familiar with common first aid principles and simple procedures (e.g., bleeding control, bandaging)

C. Limited – only know a few basic first aid facts

D. No knowledge – have never been exposed to first aid training

**Part II: Health Status**

12. Self‑assessment of physical fitness:

A. Excellent – can easily handle high‑intensity work and activities

B. Good – daily work and activities are unaffected, but high‑intensity tasks are somewhat challenging

C. Fair – easily fatigued, which affects work and activities to some extent

D. Poor – often have to reduce work or activities due to physical issues

13. Hearing condition:

A. Normal – audiometry test shows no abnormality

B. Mild abnormality – does not affect daily communication or work

C. Moderate abnormality – difficulty communicating in noisy environments

D. Severe abnormality – requires assistive devices

14. Do you currently have any chronic diseases? (Select all that apply)

A. No chronic diseases

B. Cardiovascular diseases (hypertension, hyperlipidemia, coronary heart disease, etc.)

C. Digestive system diseases (gastric ulcer, chronic hepatitis, etc.)

D. Musculoskeletal diseases (arthritis, lumbar disc herniation, etc.)

E. Metabolic diseases (diabetes, gout, etc.)

F. Other (please specify: ______)

15. Have you experienced any of the following symptoms in the past 6 months? (Select all that apply)

A. None

B. Dizziness, fatigue

C. Insomnia, excessive dreaming

D. Joint pain

E. Chest tightness, palpitations

F. Other (please specify: ______)

16. How would you describe your mental state during voyages?

A. Very good – calm, no negative emotions

B. Fairly good – occasional negative emotions but able to self‑regulate

C. Average – often anxious, lonely or other negative emotions that affect mood

D. Poor – prolonged anxiety, depression or other states that affect work and daily life

17. Have you felt depressed or anxious in the past 6 months?

A. No B. Yes

18. History of past illnesses: (Select all that apply)

A. No past illness B. Cardiovascular diseases C. Digestive system diseases D. Respiratory diseases (asthma, chronic bronchitis, etc.) E. Mental / psychological disorders (anxiety, depression, etc.) F. Other (please specify: ______)

19. Do you have any occupation‑related nutritional deficiencies? (Select all that apply)

A. None B. Beriberi (vitamin B1 deficiency) C. Mouth ulcers / angular cheilitis (vitamin B deficiency) D. Dry skin / seborrheic dermatitis E. Other (please specify: ______)

**Part III: Factors Influencing Health**

20. How regular is your daily diet on board?

A. Very regular – three meals at fixed times with fixed portions

B. Fairly regular – occasionally miss meal times

C. Not very regular – often miss meals or binge eat

D. Very irregular – chaotic meal schedule, no fixed meal times

21. What is your daily intake of vegetables and fruits?

(Note: 1 medium apple ≈ 200 g, 1 cucumber ≈ 150 g, 1 small portion of leafy vegetables ≈ 100 g)

A. 500 g or more B. 300–499 g C. 100–299 g D. Less than 100 g

22. On average, how many hours do you sleep per day on board?

A. 8 hours or more B. 6–7 hours C. 4–5 hours D. Less than 4 hours

23. How would you rate your sleep quality?

A. Very good – fall asleep quickly, sleep soundly, wake up refreshed

B. Relatively good – occasionally have difficulty falling asleep, but daytime performance is unaffected

C. Average – often have difficulty falling asleep or wake easily, mild daytime fatigue

D. Poor – chronic insomnia or light sleep, significantly affecting daytime work and life

24. Do you exercise regularly on board?

A. Yes B. No (Reason: ① lack of time ② limited space on board ③ lack of awareness ④ other: ______)

25. Do you actively pay attention to your health during voyages (e.g., self‑monitor blood pressure, pay attention to diet and nutrition, etc.)?

A. Often B. Occasionally C. Rarely D. Never

26. How are your relationships with colleagues on board?

A. Very harmonious – we help each other and get along well

B. Fairly harmonious – occasional minor frictions, but do not affect work or life

C. Average – indifferent, only work‑related communication

D. Not harmonious – frequent conflicts with colleagues

27. Do you have someone on board with whom you can confide personal matters?

A. Yes B. No

28. How supportive do you feel your family is of your job?

A. Very supportive – give full understanding and encouragement

B. Fairly supportive – sometimes worry but respect my choice

C. Neutral – attitude not clear

D. Not very supportive – hope I would change jobs

29. When you encounter psychological distress on board, to whom do you turn for help? (Select all that apply)

A. Colleagues B. Family C. On‑board psychological counseling service D. Bear it alone E. Other (please specify: ______)

30. Do you experience seasickness during voyages?

A. Never seasick

B. Mild – occasional discomfort, does not affect work or daily life

C. Moderate – discomfort at the beginning of a voyage but gradually adapts

D. Severe – frequent nausea and vomiting, affecting work and daily life

31. How would you rate the risk level of your work position?

A. Low risk (e.g., logistics, clerical, cook – no direct safety hazards)

B. Medium risk (e.g., ordinary crew, watchkeepers – minor safety hazards requiring routine protection)

C. High risk (e.g., deck work, engine room operations, working aloft or over the side – higher safety hazards requiring specialized protection)

32. What is the typical duration of a single voyage/sea tour (time on board)?

A. Less than 6 months B. 6–12 months C. 12–18 months D. More than 18 months

33. What is the longest continuous working period you have ever had? _____ hours

34. What is your work shift pattern?

A. Fixed day shift B. Fixed night shift C. Rotating shift D. No fixed shift – on‑call duty as required

35. How satisfied are you with your current workload on board?

A. Very satisfied – workload is moderate, and I can accomplish tasks well

B. Fairly satisfied – somewhat tiring but acceptable

C. Average – workload is heavy, and I often feel exhausted

D. Unsatisfied – workload is excessive, severely affecting physical and mental health

36. How do you feel the working environment on board (noise, temperature, humidity, vibration, etc.) affects your health?

A. Almost no effect – working environment is good

B. Some effect, but not severe – I can adapt

C. Considerable effect – often feel physical discomfort

D. Great effect – often feel obvious discomfort

37. How satisfied are you with the accommodation conditions on board?

A. Very satisfied – spacious, well‑equipped, clean and tidy

B. Fairly satisfied – basic living needs are met, although conditions are average

C. Average – cramped, outdated facilities, not very comfortable

D. Unsatisfied – very poor accommodation, severely affecting rest

38. Are there sufficient recreational facilities and activities on board to help relieve stress?

A. Yes – abundant facilities and regular activities, very helpful for stress relief

B. Fairly sufficient – some basic facilities and activities, offer moderate relaxation

C. Average – few facilities and activities, hard to spend leisure time

D. Almost none – very boring outside working hours

39. Which of the following best describes the medical care provision on your vessel?

A. Full‑time ship’s doctor with well‑equipped medical facilities and medicines

B. No ship's doctor, but prompt access to tele‑medical guidance/emergency support

C. Only basic first‑aid medicines available, no dedicated medical staff

D. Very limited medical supplies, virtually no medical care

40. Are you able to make voice calls normally while on board?

A. Can call anytime with good connection B. Can call during scheduled times

C. Occasionally can connect, signal unstable D. Basically unable to make voice calls

41. Are you familiar with the national or company policies regarding seafarers’ health protection?

A. Very familiar B. Somewhat familiar C. Not very familiar D. Not familiar at all

42. Does your company arrange regular health check‑ups for seafarers?

A. Yes – check‑ups are arranged before and after each embarkation/disembarkation

B. Yes – about once a year on average

C. Occasionally (once every few years)

D. Never arranged

43. How satisfied are you with the health check‑ups arranged by your company?

A. Very satisfied – comprehensive items, standard procedures, timely feedback

B. Fairly satisfied – basic items meet needs, minor issues acceptable

C. Average – items not comprehensive, procedures cumbersome, feedback delayed

D. Unsatisfied – check‑ups are merely a formality, with no real value

44. Would you be willing to participate in health intervention activities organized by your company (e.g., health talks, fitness guidance, psychological counseling, etc.)?

A. Very willing B. Willing C. Not sure D. Unwilling

**Part IV: Health Management Needs**

45. What aspects of seafarers' health management would you like the company/government to strengthen? (Select all that apply)

A. Improve the diet on board (increase supply of fresh fruits, vegetables and high‑quality protein)

B. Strengthen occupational health training (first aid, prevention of occupational diseases, etc.)

C. Upgrade on‑board medical facilities and medicine supplies

D. Improve mental health support systems (provide psychological counselors, open helplines)

E. Optimize voyage scheduling, reduce the duration of each single assignment

F. Establish seafarer health records for dynamic health management

G. Enhance communication with seafarers’ families to build a family‑support system

H. Other (please specify: ______)

End of questionnaire. Thank you for your cooperation and support!
