# Supplementary material for: Health-related factors among seafarers based on the health ecology model: a cross-sectional study
Source: Front Public Health. 2026 Oct 15;14:1942282. doi: 10.3389/fpubh.2026.1942282 (PMC13716717; doi:10.3389/fpubh.2026.1942282)
Supplement: Supplementary file 2 [file Supplementary_file_2.docx]

**Appendix 2: Scoring Rules for Variables**

**Appendix Table 1 – Scoring Rules for Dependent Variables**

| Item | Content | Scoring Rules |
| --- | --- | --- |
| Q12 | Self‑assessed physical fitness | Excellent = 4, Good = 3, Fair = 2, Poor = 1 |
| Q13 | Hearing condition | Normal = 4, Mild abnormality = 3, Moderate abnormality = 2, Severe abnormality = 1 |
| Q14 | Chronic diseases (multiple choice) | 0 items selected = 4, 1 item = 2, ≥2 items = 0 |
| Q15 | Symptoms in the past 6 months (multiple choice) | 0 items selected = 4, 1 item = 2, ≥2 items = 0 |
| Q16 | Mental state during voyages | Very good = 4, Fairly good = 3, Average = 2, Poor = 1 |
| Q17 | Depressed or anxious in the past 6 months | No = 4, Yes = 1 |
| Q18 | History of past illnesses (multiple choice) | 0 items selected = 4, 1 item = 2, ≥2 items = 0 |
| Q19 | Nutritional deficiencies (multiple choice) | 0 items selected = 4, 1 item = 2, ≥2 items = 0 |

Scoring instructions:

- Total health score (range 2–32) = Q12 + Q13 + Q14 + Q15 + Q16 + Q17 + Q18 + Q19

- Physical health dimension score (range 0–24) = Q12 + Q13 + Q14 + Q15 + Q18 + Q19

- Mental health dimension score (range 2–8) = Q16 + Q17

**Appendix Table 2 – Scoring Rules for Independent Variables: Behavioral Characteristics**

| Item | Content | Scoring Rules |
| --- | --- | --- |
| Q20 | Regularity of daily diet | Very regular = 4, Fairly regular = 3, Not very regular = 2, Very irregular = 1 |
| Q21 | Daily vegetable and fruit intake | ≥500 g = 4, 300–499 g = 3, 100–299 g = 2, <100 g = 1 |
| Q22 | Average daily sleep duration | ≥8 hours = 4, 6–7 hours = 3, 4–5 hours = 2, <4 hours = 1 |
| Q23 | Sleep quality | Very good = 4, Relatively good = 3, Average = 2, Poor = 1 |
| Q24 | Regular physical exercise | Yes = 1, No = 0 |
| Q25 | Health‑attention behavior | Often = 4, Occasionally = 3, Rarely = 2, Never = 1 |

Scoring instructions:

Behavioral characteristics score (range 0–21) = Q20 + Q21 + Q22 + Q23 + Q24 + Q25

**Appendix Table 3 – Scoring Rules for Independent Variables: Interpersonal Networks**

| Item | Content | Scoring Rules |
| --- | --- | --- |
| Q26 | Relationships with colleagues | Very harmonious = 4, Fairly harmonious = 3, Average = 2, Not harmonious = 1 |
| Q27 | Having a confidant on board | Yes = 1, No = 0 |
| Q28 | Family support for the job | Very supportive = 4, Fairly supportive = 3, Neutral = 2, Not very supportive = 1 |
| Q29 | Sources of help when distressed (multiple choice) | Count of selected options A (colleagues) / B (family) / C (counseling service): 0 selected = 0, 1 selected = 1, 2 selected = 2, 3 selected = 3 |

Scoring instructions:

Interpersonal network score (range 0–12) = Q26 + Q27 + Q28 + Q29

**Appendix Table 4 – Scoring Rules for Independent Variables: Living and Working Conditions**

| Item | Content | Scoring Rules |
| --- | --- | --- |
| Q30 | Seasickness experience | Never = 4, Mild = 3, Moderate = 2, Severe = 1 |
| Q31 | Job risk level | Low risk = 3, Medium risk = 2, High risk = 1 |
| Q32 | Typical duration of a single voyage | <6 months = 4, 6–12 months = 3, 12–18 months = 2, >18 months = 1 |
| Q33 | Longest continuous working period | ≤8 h = 4, 9–10 h = 3, 11–12 h = 2, ≥13 h = 1 |
| Q34 | Work shift pattern | Fixed shift = 1, Non‑fixed shift = 0 |
| Q35 | Satisfaction with workload | Very satisfied = 4, Fairly satisfied = 3, Average = 2, Unsatisfied = 1 |
| Q36 | Impact of the working environment | Almost no effect = 4, Some effect = 3, Considerable effect = 2, Great effect = 1 |
| Q37 | Satisfaction with accommodation | Very satisfied = 4, Fairly satisfied = 3, Average = 2, Unsatisfied = 1 |
| Q38 | Adequacy of recreational facilities | Abundant = 4, Fairly sufficient = 3, Average = 2, Almost none = 1 |
| Q39 | Medical care provision | Better (ship’s doctor or tele‑guidance available) = 1, Poorer (only basic medicines / very limited supplies) = 0 |
| Q40 | Voice call conditions | Can call anytime = 4, Scheduled times = 3, Occasionally = 2, Basically unable = 1 |

Scoring instructions:

Living and working conditions score (range 0–37) = Q30 + Q31 + Q32 + Q33 + Q34 + Q35 + Q36 + Q37 + Q38 + Q39 + Q40

**Appendix Table 5 – Scoring Rules for Independent Variables: Policy Environment**

| Item | Content | Scoring Rules |
| --- | --- | --- |
| Q41 | Awareness of health protection policies | Very familiar = 4, Somewhat familiar = 3, Not very familiar = 2, Not at all familiar = 1 |
| Q42 | Regular health check‑up arrangement | Arranged before/after each embarkation = 4, About once a year = 3, Occasionally = 1, Never = 0 |
| Q43 | Satisfaction with check‑ups | Very satisfied = 4, Fairly satisfied = 3, Average = 2, Unsatisfied = 1 |
| Q44 | Willingness to participate in health interventions | Very willing = 4, Willing = 3, Not sure = 2, Unwilling = 1 |

Scoring instructions:

Policy environment score (range 1–16) = Q41 + Q42 + Q43 + Q44

**Summary of Scoring Ranges**

| Variable Type | Dimension / Variable | Calculation Formula | Theoretical Range |
| --- | --- | --- | --- |
| Dependent | Total health score | Q12+Q13+Q14+Q15+Q16+Q17+Q18+Q19 | 2~32 |
|  | Physical health dimension | Q12+Q13+Q14+Q15+Q18+Q19 | 0~24 |
|  | Mental health dimension | Q16+Q17 | 2~8 |
| Independent | Behavioral characteristics | Q20+Q21+Q22+Q23+Q24+Q25 | 0~21 |
|  | Interpersonal network | Q26+Q27+Q28+Q29 | 0~12 |
|  | Living and working conditions | Q30+Q31+Q32+Q33+Q34+Q35+Q36+Q37+Q38+Q39+Q40 | 0~37 |
|  | Policy environment | Q41+Q42+Q43+Q44 | 1~16 |

Note on Q45: Q45 (Health management needs) is not included in the regression analysis; it will only be reported descriptively in the results section. Therefore, it is not listed in the scoring tables. For Q45, a bar chart will be used to present the selection rate for each option.
