# Supplementary material for: Health-related factors among seafarers based on the health ecology model: a cross-sectional study
Source: Front Public Health. 2026 Oct 15;14:1942282. doi: 10.3389/fpubh.2026.1942282 (PMC13716717; doi:10.3389/fpubh.2026.1942282)
Supplement: Supplementary file 3 [file Table_1.docx]

**Supplementary Table 1 – Correlation Analysis of Scores of Health‑Influencing Factor Dimensions (*n* = 106)**

| Dimension | Behavioral characteristics | Interpersonal network | Working conditions | Policy environment |
| --- | --- | --- | --- | --- |
| Behavioral characteristics | 1 |  |  |  |
| Interpersonal network | 0.563** | 1 |  |  |
| Working conditions | 0.727** | 0.547** | 1 |  |
| Policy environment | 0.683** | 0.566** | 0.686** | 1 |

Note: ***P*<0.01
