# Supplementary material for: Health-related factors among seafarers based on the health ecology model: a cross-sectional study
Source: Front Public Health. 2026 Oct 15;14:1942282. doi: 10.3389/fpubh.2026.1942282 (PMC13716717; doi:10.3389/fpubh.2026.1942282)
Supplement: Supplementary file 4 [file Table_2.docx]

**Supplementary Table 2 – Multiple Linear Regression Analysis of Factors Influencing Physical Health Dimension Score of Seafarers (*n*=106)**

| Variable | *β* | Std. Error | 95%CI | *Beta* | *t* | *P* | Tolerance | VIF |
| --- | --- | --- | --- | --- | --- | --- | --- | --- |
| Constant | 12.385 | 1.988 | 8.44–16.33 |  | 6.231 | ＜0.001 |  |  |
| Age | -0.001 | 0.020 | -0.04–0.040 | -0.006 | -0.073 | 0.942 | 0.770 | 1.298 |
| Educational level | 0.015 | 0.241 | −0.46–0.49 | 0.005 | 0.062 | 0.951 | 0.706 | 1.417 |
| First‑aid knowledge | -0.375 | 0.360 | −1.09–0.34 | -0.085 | -1.039 | 0.301 | 0.714 | 1.400 |
| Behavioral characteristics score | 0.260 | 0.112 | 0.04–0.48 | 0.267 | 2.326 | 0.022 | 0.366 | 2.730 |
| Interpersonal network score | 0.115 | 0.158 | −0.20–0.43 | 0.065 | 0.728 | 0.468 | 0.600 | 1.666 |
| Living and working conditions score | -0.049 | 0.059 | −0.17–0.07 | -0.099 | -0.832 | 0.407 | 0.342 | 2.928 |
| Policy environment score | 0.526 | 0.098 | 0.33–0.72 | 0.591 | 5.355 | <0.001 | 0.396 | 2.524 |

Note: The dependent variable is the physical health dimension score. *β* (B) is the unstandardized regression coefficient, and *Beta* is the standardized regression coefficient; CI is the 95% confidence interval for *β;* VIF stands for the variance inflation factor. Model summary: *F*(7,98) = 15.551, *P* < 0.001, *R²* = 0.526, adjusted *R²* = 0.492, Durbin‑Watson value = 1.762.
