# Supplementary material for: Health-related factors among seafarers based on the health ecology model: a cross-sectional study
Source: Front Public Health. 2026 Oct 15;14:1942282. doi: 10.3389/fpubh.2026.1942282 (PMC13716717; doi:10.3389/fpubh.2026.1942282)
Supplement: Supplementary file 5 [file Table_3.docx]

**Supplementary Table 3 – Multiple Linear Regression Analysis of Factors Influencing Mental Health Dimension Score of Seafarers (*n*=106)**

| Variable | *β* | Std. Error | 95% CI | *Beta* | *t* | *P* | Tolerance | VIF |
| --- | --- | --- | --- | --- | --- | --- | --- | --- |
| Constant | 0.743 | 1.052 | −1.35–2.83 |  | 0.707 | 0.481 |  |  |
| Age | 0.036 | 0.011 | 0.015–0.057 | 0.279 | 3.366 | 0.001 | 0.770 | 1.298 |
| Educational level | 0.031 | 0.127 | −0.22–0.28 | 0.021 | 0.242 | 0.809 | 0.706 | 1.417 |
| First‑aid knowledge | 0.076 | 0.191 | −0.30–0.46 | 0.034 | 0.398 | 0.691 | 0.714 | 1.400 |
| Behavioral characteristics score | 0.083 | 0.059 | −0.03–0.20 | 0.169 | 1.408 | 0.162 | 0.366 | 2.730 |
| Interpersonal network score | 0.185 | 0.084 | 0.02–0.35 | 0.207 | 2.207 | 0.030 | 0.600 | 1.666 |
| Living and working conditions score | -0.043 | 0.031 | −0.10–0.02 | -0.171 | -1.370 | 0.174 | 0.342 | 2.928 |
| Policy environment score | 0.199 | 0.052 | 0.10–0.30 | 0.444 | 3.840 | <0.001 | 0.396 | 2.524 |

Note: The dependent variable is the mental health dimension score of seafarers. *β* (B) is the unstandardized regression coefficient, and *Beta* is the standardized regression coefficient; CI is the 95% confidence interval for *β*; VIF stands for the variance inflation factor. Model summary: *F*(7,98) = 12.969, *P* < 0.001, *R²* = 0.481, adjusted *R²* = 0.444, Durbin‑Watson value = 2.029.
