# Supplementary material for: Health-related factors among seafarers based on the health ecology model: a cross-sectional study
Source: Front Public Health. 2026 Oct 15;14:1942282. doi: 10.3389/fpubh.2026.1942282 (PMC13716717; doi:10.3389/fpubh.2026.1942282)
Supplement: Supplementary file 6 [file Table_4.docx]

**Supplementary Table 4 – Coefficient Stability in Leave-One-Dimension-Out Models (*n*=106)**

| Omitted dimension | Behavioral characteristics *β* | Interpersonal network *β* | Living/working conditions *β* | Policy environment *β* | *Model R^2^* |
| --- | --- | --- | --- | --- | --- |
| None (full model, 7 predictors) | 0.260 | 0.125 | -0.137 | 0.602 | 0.603 |
| Behavioral characteristics | — | 0.162 | -0.043 | 0.672 | 0.578 |
| Interpersonal network | 0.289 | — | -0.110 | 0.632 | 0.593 |
| Living/working conditions | 0.214 | 0.109 | — | 0.560 | 0.596 |
| Policy environment | 0.437 | 0.221 | 0.074 | — | 0.459 |

Note: Standardized regression coefficients (*β*) of the four ecological dimension scores in the full multivariable model and in models omitting one dimension at a time. Age, educational level, and first-aid knowledge were retained as control variables in all models.
