# Supplementary material for: Health-related factors among seafarers based on the health ecology model: a cross-sectional study
Source: Front Public Health. 2026 Oct 15;14:1942282. doi: 10.3389/fpubh.2026.1942282 (PMC13716717; doi:10.3389/fpubh.2026.1942282)
Supplement: Supplementary file 7 [file Table_5.docx]

**Supplementary Table 5 – Commonality Analysis of the Four Ecological Dimensions (*n*=106)**

| Dimension | Zero-order *R²* (total contribution) | Unique contribution | Common/shared contribution |
| --- | --- | --- | --- |
| Behavioral characteristics | 0.414 | 0.028 | 0.385 |
| Interpersonal network | 0.278 | 0.008 | 0.270 |
| Living/working conditions | 0.295 | 0.004 | 0.291 |
| Policy environment | 0.547 | 0.135 | 0.412 |

Note: The joint *R²* of the four dimensions was 0.593. Unique contribution = joint *R²* − *R²* of the model excluding that dimension; common/shared contribution = zero-order *R²* − unique contribution. The summed zero-order contributions (1.533) exceeded the joint *R²* by 0.940, indicating that 61% of the total explained variance was overlapping variance shared among dimensions and cannot be attributed to any single dimension.
