# Supplementary material for: Health-related factors among seafarers based on the health ecology model: a cross-sectional study
Source: Front Public Health. 2026 Oct 15;14:1942282. doi: 10.3389/fpubh.2026.1942282 (PMC13716717; doi:10.3389/fpubh.2026.1942282)
Supplement: Supplementary file 8 [file Table_6.docx]

**Supplementary Table 6 – Psychometric Evaluation of the Self-rated Health Status Scale (*n* = 106)**

| **Item** | **EFA loading (one factor)** | **Communality (*h*²)** | **Corrected item-total correlation** | ***α* if item deleted** |
| --- | --- | --- | --- | --- |
| Q12 Self-rated physical fitness | 0.620 | 0.384 | 0.545 | 0.766 |
| Q13 Hearing status | 0.444 | 0.197 | 0.382 | 0.792 |
| Q14 Chronic diseases | 0.422 | 0.178 | 0.391 | 0.792 |
| Q15 Somatic symptoms (past 6 months) | 0.582 | 0.339 | 0.537 | 0.764 |
| Q16 Mental state during voyages | 0.815 | 0.664 | 0.698 | 0.743 |
| Q17 Depressed/anxious (past 6 months) | 0.618 | 0.382 | 0.507 | 0.772 |
| Q18 Past medical history | 0.525 | 0.275 | 0.459 | 0.777 |
| Q19 Nutritional deficiencies | 0.669 | 0.447 | 0.621 | 0.749 |

**Note:** EFA by principal axis factoring. The correlation matrix was factorable (Kaiser-Meyer-Olkin measure = 0.804; Bartlett's test of sphericity: *χ*²(28) = 239.013, *P* < 0.001); parallel analysis supported a single factor, which explained 35.8% of the total variance. McDonald's *ω* = 0.811; Cronbach's *α* = 0.793. A two-factor exploratory solution (principal axis factoring with promax rotation) converged (factor correlation = 0.70) but did not reproduce the intended six physical + two mental item partition. Confirmatory factor analysis comparison: one-factor model *χ*² (20) = 42.742, CFI = 0.897, TLI = 0.856, RMSEA = 0.104; two-factor model *χ*² (19) = 35.565, CFI = 0.925, TLI = 0.890, RMSEA = 0.091, SRMR = 0.064, factor correlation = 0.83. Sensitivity analyses: the total score correlated at Spearman r = 0.9998 with a linearly scored version and r = 0.9968 with a dichotomous version of the multiple-choice items.
