# Supplementary material for: Health-related factors among seafarers based on the health ecology model: a cross-sectional study
Source: Front Public Health. 2026 Oct 15;14:1942282. doi: 10.3389/fpubh.2026.1942282 (PMC13716717; doi:10.3389/fpubh.2026.1942282)
Supplement: Supplementary file 9 [file Table_7.docx]

**Supplementary Table 7 – Factor Analysis of the Four Reflective Dimensions of the Health Influencing Factors Scale (*n* = 106)**

**Panel A. Dimension-level summary**

| **Dimension** | **Items (*n*)** | **KMO** | **Bartlett χ² (df)** | ***P*** | **Variance explained (%)** | **McDonald's *ω*** | **Cronbach's *α*** |
| --- | --- | --- | --- | --- | --- | --- | --- |
| Behavioral characteristics | 6 | 0.773 | 164.717 (15) | <0.001 | 39.0 | 0.790 | 0.768 |
| Interpersonal network | 4 | 0.718 | 59.363 (6) | <0.001 | 34.8 | 0.675 | 0.643 |
| Living/working conditions | 11 | 0.818 | 441.080 (55) | <0.001 | 35.7 | 0.844 | 0.820 |
| Policy environment | 4 | 0.815 | 215.728 (6) | <0.001 | 64.5 | 0.878 | 0.869 |

**Panel B. First-factor loadings (absolute values)**

| **Dimension** | **Item** | **Loading** |
| --- | --- | --- |
| Behavioral characteristics | Diet regularity | 0.569 |
|  | Fruit/vegetable intake | 0.629 |
|  | Sleep duration | 0.533 |
|  | Sleep quality | 0.688 |
|  | Physical exercise | 0.525 |
|  | Health attention | 0.765 |
| Interpersonal network | Colleague relations | 0.623 |
|  | Confidant on board | 0.607 |
|  | Family support | 0.676 |
|  | Coping when distressed | 0.420 |
| Living/working conditions | Seasickness experience | 0.274 |
|  | Job risk level | 0.709 |
|  | Voyage duration | 0.218 |
|  | Longest continuous work period | 0.525 |
|  | Work shift type | 0.411 |
|  | Workload satisfaction | 0.708 |
|  | Work environment impact | 0.769 |
|  | Accommodation satisfaction | 0.877 |
|  | Recreational facilities | 0.662 |
|  | Medical care provision | 0.448 |
|  | Voice call conditions | 0.594 |
| Policy environment | Health policy awareness | 0.758 |
|  | Regular check-up arrangement | 0.763 |
|  | Check-up satisfaction | 0.897 |
|  | Intervention willingness | 0.785 |

**Note:** Principal axis factoring with squared multiple correlations as initial communality estimates; unrotated one-factor solution (factor sign arbitrary; absolute values shown). Bartlett's test of sphericity was significant for all four dimensions (all *P* < 0.001). The interpersonal network dimension had the lowest internal consistency (*α* = 0.643; *ω* = 0.675). For the living/working conditions dimension, the second eigenvalue marginally exceeded the random-data criterion, but the two-factor solution was not interpretable (second-factor loadings all < 0.13); the dimension was therefore retained as a composite index.
