# Supplementary material for: Health-related factors among seafarers based on the health ecology model: a cross-sectional study
Source: Front Public Health. 2026 Oct 15;14:1942282. doi: 10.3389/fpubh.2026.1942282 (PMC13716717; doi:10.3389/fpubh.2026.1942282)
Supplement: Supplementary file 10 [file Supplementary_file_3.doc]

STROBE Statement—Checklist of items that should be included in reports of ***cross-sectional studies***

|  | Item No | Recommendation | **Description** |
| --- | --- | --- | --- |
| **Title and abstract** | 1 | (*a*) Indicate the study’s design with a commonly used term in the title or the abstract | The title explicitly states “cross-sectional survey”; the abstract specifies “using convenience sampling... cross-sectional study”. |
|  |  | (*b*) Provide in the abstract an informative and balanced summary of what was done and what was found | The abstract summarises: objectives (based on the health ecological model), methods (convenience sampling, 106 cases, multiple linear regression), main findings (total health score 29.65±3.62, policy environment *β*=0.602, behavioral characteristics *β*=0.260), and conclusions. |
| Introduction | | |  |
| Background/rationale | 2 | Explain the scientific background and rationale for the investigation being reported. | Paragraphs 1-2 of the Introduction describe the scale of the shipping industry, health risks, and limitations of previous studies; paragraph 3 introduces the health ecological model; paragraph 4 describes the current health infrastructure and policy context in the Zhoushan area. |
| Objectives | 3 | State-specific objectives, including any prespecified hypotheses | The end of paragraph 4 and paragraph 5 of the Introduction clearly state the objective: “to quantify the relative contributions of each dimension within the five-level framework of the health ecological model”, and propose three specific hypotheses. |
| Methods | | |  |
| Study design | 4 | Present key elements of the study design early in the paper | Section 2.1 explicitly states: “This is a cross-sectional study and is reported in accordance with the STROBE Statement.” |
| Setting | 5 | Describe the setting, locations, and relevant dates, including periods of recruitment, exposure, follow-up, and data collection. | Section 2.2 states that the study was conducted at maritime service counters in Zhoushan City, with data collection from 1 March to 30 June 2026. |
| Participants | 6 | (*a*) Give the eligibility criteria, and the sources and methods of selection of participants | Section 2.2 specifies inclusion criteria (active seafarers, with seagoing experience, voluntarily participating), exclusion criteria (incomplete questionnaires, completion time <180 seconds), and sampling method (convenience sampling at maritime service counters). |
| Variables | 7 | Clearly define all outcomes, exposures, predictors, potential confounders, and effect modifiers. Give diagnostic criteria, if applicable. | Section 2.4 defines: dependent variable = total health score (comprising 6 physical health items and 2 mental health items); independent variables = five dimensions (individual characteristics, behavioral characteristics, interpersonal network, living and working conditions, policy environment); control variables = age, educational level, and first‑aid knowledge level. |
| Data sources/ measurement | 8* | For each variable of interest, give sources of data and details of methods of assessment (measurement). Describe the comparability of assessment methods if there is more than one group. | Section 2.4 and Appendix 2 detail the sources and scoring rules for each variable (ordinal, dichotomous, and multiple-choice items). This cross‑sectional study does not involve group comparisons, so comparability of assessment methods is not applicable. |
| Bias | 9 | Describe any efforts to address potential sources of bias | The “Limitations” section acknowledges potential bias from convenience sampling and self-report; Section 2.2 explains that the sampling site was chosen to cover different routes, ranks, and companies to reduce selection bias. |
| Study size | 10 | Explain how the study size was arrived at | Section 2.3 explains that, based on the empirical rule for multiple linear regression (10–15 times the number of independent variables), with 7 independent variables, a minimum of 105 cases was required (15× rule); the final sample was 106. Section 2.3 additionally reports a sensitivity (post hoc) power analysis (minimum detectable effect: standardized *β* ≈ 0.27 per predictor at 80% power). |
| Quantitative variables | 11 | Explain how quantitative variables were handled in the analyses. If applicable, describe which groupings were chosen and why | Section 2.6 states that continuous variables were first tested for normality using the Kolmogorov–Smirnov test; normally distributed variables are presented as mean ± SD; non-normally distributed variables as median (IQR); and categorical variables as frequencies and percentages. The boundaries for categorical variables are shown in Table 1 (educational level, years of service, first-aid knowledge level, etc.). |
| Statistical methods | 12 | (*a*) Describe all statistical methods, including those used to control for confounding | Section 2.6 reports: univariate analyses (*t*-test, ANOVA/Welch correction, Pearson correlation, with screening criterion *P*<0.10); multiple linear regression (forced entry); collinearity diagnostics (VIF<10); residual independence test (Durbin–Watson). |
|  |  | (*b*) Describe any methods used to examine subgroups and interactions | Section 2.6 states that separate regression analyses were performed using the same set of independent variables, with physical health dimension score and mental health dimension score as dependent variables, respectively (results in Supplementary Tables 2 and 3). |
|  |  | (*c*) Explain how missing data were addressed | Section 2.3 indicates that 1 invalid questionnaire was excluded (incomplete), and the final analysis included 106 complete questionnaires; no variables had missing data. |
|  |  | (*d*) If applicable, describe analytical methods taking account of the sampling strategy | This study used convenience sampling without complex weighting; it is not applicable. |
|  |  | (*e*) Describe any sensitivity analyses | Sensitivity analyses were performed: robustness of the total score to alternative scoring of the multiple-choice items (Section 2.4.2), a leave-one-dimension-out coefficient stability analysis and a commonality analysis (Section 3.4; Supplementary Tables 4 and 5), and a decomposition of the policy environment dimension (Discussion). |
| Results | | |  |
| Participants | 13* | (a) Report numbers of individuals at each stage of study—e.g., numbers potentially eligible, examined for eligibility, confirmed eligible, included in the study, completing follow-up, and analyzed | Section 2.3 reports: 109 questionnaires distributed, 107 returned (response rate 98.17%), 1 invalid excluded, final 106 valid (valid response rate 97.25%)—no follow-up stage in this cross-sectional study. |
|  |  | (b) Give reasons for non-participation at each stage | The reason for exclusion (1 incomplete) is given; the number and reasons for refusal to participate are not reported in the manuscript. |
|  |  | (c) Consider the use of a flow diagram | As this is a single-time cross-sectional survey, no flow diagram was used (could be noted as “not applicable”). |
| Descriptive data | 14* | (a) Give characteristics of study participants (e.g., demographic, clinical, social) and information on exposures and potential confounders | Table 1 in Section 3.1 reports all demographic characteristics: age, educational level, years of service, job category, route area, family history, smoking, alcohol consumption, first-aid knowledge level, etc. |
|  |  | (b) Indicate the number of participants with missing data for each variable of interest | Section 2.3 states that the final analysis included 106 complete questionnaires; no missing data for any variable. |
| Outcome data | 15* | Report the number of outcome events or summary measures | Table 2 in Section 3.2 reports: total health score (29.65±3.62), physical health dimension score (22.27±2.67), mental health dimension score (7.38±1.35), and scores for each influencing factor dimension. |
| Main results | 16 | (*a*) Give unadjusted estimates and, if applicable, confounder-adjusted estimates and their precision (eg, 95% confidence interval). Make clear which confounders were adjusted for and why they were included | Table 3 presents unadjusted results (univariate analyses, with effect sizes); Table 4 presents adjusted results from multiple regression (B, standardized Beta, *P* values, VIF); 95% CIs for the dimension-specific regressions are reported in Supplementary Tables 2 and 3. |
|  |  | (*b*) Report category boundaries when continuous variables were categorized | Table 1 reports the boundaries for categorical variables (educational level, years of service, first-aid knowledge level, etc.); age was treated as a continuous variable. |
|  |  | (*c*) If relevant, consider translating estimates of relative risk into absolute risk for a meaningful time period | Not applicable for cross‑sectional studies. |
| Other analyses | 17 | Report other analyses done—e.g., analyses of subgroups and interactions, and sensitivity analyses | Section 3.5 and Supplementary Tables 2 and 3 report separate regression analyses for physical and mental health dimension scores; Section 3.4 and Supplementary Tables 4 and 5 report the leave-one-dimension-out and commonality analyses. |
| Discussion | | |  |
| Key results | 18 | Summarise key results with reference to study objectives | The first paragraph of the Discussion summarises that the total health score was close to the theoretical maximum, and that policy environment and behavioral characteristics were the main influencing factors. |
| Limitations | 19 | Discuss limitations of the study, taking into account sources of potential bias or imprecision. Discuss both the direction and the magnitude of any potential bias. | The "Limitations" section lists nine limitations, including the limited statistical power and minimum detectable effect (with the null results being inconclusive rather than confirmatory), the triple selection bias of the sample, the cross-sectional design, self-report bias, the all-male sample, the relatively low Cronbach's *α* for interpersonal networks, skewed variable distributions, the absence of a control group, and the preliminary psychometric support of the self-developed scales (including the self-reported, perception-based measurement of the policy environment). |
| Interpretation | 20 | Give a cautious overall interpretation of results, considering objectives, limitations, multiplicity of analyses, results from similar studies, and other relevant evidence. | The Discussion (paragraphs 2—8) interprets the results within the health ecological model, compares them with previous studies (Jiang et al., Zhao et al., Kerkamm et al., Strukcinskiene et al.), and offers cautious conclusions. |
| Generalisability | 21 | Discuss the generalisability (external validity) of the study results | The first and fourth limitations in the Discussion point out that the sample was predominantly coastal‑route and management-level, so generalization to ocean-going seafarers and other regions requires caution. |
| Other information | | |  |
| Funding | 22 | Give the source of funding and the role of the funders for the present study and, if applicable, for the original study on which the present article is based. | The footnote on the title page states the funding sources: Zhejiang Provincial Department of Science and Technology Soft Science Research Project (No. 2026C25050) and Zhoushan Science and Technology Bureau Applied Technology Research Project (No. 2026C31049). |

Note: Items marked with an asterisk (*) (Items 8, 13, 14, 15) are reported as described above for cross-sectional studies. If this checklist is to be uploaded as a supplementary file, you may add an extra column for "Page/Section" to indicate the exact locations in your manuscript.
